# Does Routine Anti-Osteoporosis Medication Lower the Risk of Fractures in Male Subjects? An Updated Systematic Review With Meta-Analysis of Clinical Trials

**DOI:** 10.3389/fphar.2019.00882

**Published:** 2019-08-09

**Authors:** Ling-Feng Zeng, Bi-Qi Pan, Gui-Hong Liang, Ming-Hui Luo, Ye Cao, Da Guo, Hong-Yun Chen, Jian-Ke Pan, He-Tao Huang, Qiang Liu, Zi-Tong Guan, Yan-Hong Han, Di Zhao, Jin-Long Zhao, Sen-Rong Hou, Ming Wu, Jiong-Tong Lin, Jia-Hui Li, Wei-Xiong Liang, Ai-Hua Ou, Qi Wang, Wei-Yi Yang, Jun Liu

**Affiliations:** ^1^The 2nd Affiliated Hospital of Guangzhou University of Chinese Medicine (Guangdong Provincial Hospital of Chinese Medicine), Guangzhou, China; ^2^Bone and Joint Research Team of Degeneration and Injury, Guangdong Provincial Academy of Chinese Medical Sciences, Guangzhou, China; ^3^The Second Clinical College of Guangzhou University of Chinese Medicine, Guangzhou, China; ^4^Department of Traditional Chinese Medicine, Guangdong Women and Children Hospital, Guangzhou, China; ^5^Department of Clinical Research/National Clinical Trials Institute, Sun Yat-sen University Cancer Center, Guangzhou, China; ^6^World Federation of Chinese Medicine Societies, Beijing, China

**Keywords:** anti-osteoporosis medication, routine therapy, osteoporotic fracture, clinical trials, risk reduction, literature review

## Abstract

**Background:** Several epidemiological articles have reported the correlations between anti-osteoporosis medication and the risks of fractures in male and female subjects, but the specific efficacy of anti-osteoporosis medication for male subjects remains largely unexplored.

**Objective:** The aim of this study was to evaluate the correlation between anti-osteoporosis medication and the risk of fracture in relation to low bone mass [including outcomes of osteoporosis, fracture, and bone mineral density (BMD) loss] in male subjects analyzed in studies within the updated literature.

**Methods:** Randomized controlled trials (RCTs) that analyzed the effectiveness of a treating prescription for male subjects with osteoporosis (or low BMD) and that focused on the outcomes of fracture were included. Relevant studies from Embase, Web of Science, PubMed, and Chinese database of CNKI were retrieved from inception to January 30th, 2019. Two staff members carried out the eligibility assessment and data extraction. The discrepancies were settled by consultation with another researcher. We calculated the pooled relative risks (RRs) based on 95% confidence intervals (CIs).

**Results:** Twenty-seven documents (28 studies) with 5,678 subjects were identified. For the category of bisphosphonates, significant results were observed in pooled analyses for decreased risk of the vertebral fracture domain (RR, 0.44 [95% CI, 0.31–0.62]), nonvertebral fracture domain (RR, 0.63 [95% CI, 0.46–0.87]), and clinical fracture domain (RR, 0.59 [95% CI, 0.48–0.72]) compared with those of controls. Participants with bisphosphonates had a 56% (95% CI = 38–69%) lower risk of vertebral fractures, 37% (95% CI = 13–54%) lower risk of nonvertebral fractures, and 41% (95% CI = 28–52%) lower risk of clinical fractures. Furthermore, meta-analyses also demonstrated a decreased risk of the vertebral fracture domain *via* treatment with risedronate (RR, 0.45 [95% CI, 0.28–0.72]) and alendronate (RR, 0.41 [95% CI, 0.23–0.74]), but not with calcitriol, calcitonin, denosumab, ibandronate, monofluorophosphate, strontium ranelate, teriparatide, or zoledronic acid, compared with that of controls.

**Conclusions:** This systematic review confirms that bisphosphonates were connected with a decreased risk of vertebral fractures, nonvertebral fractures, and clinical fractures for male subjects with osteoporosis. Future research is needed to further elucidate the role of nonbisphosphonates in treating fractures of osteoporosis subjects.

## Introduction

Recent evidence has found that 2 million male subjects in the United States have been affected by osteoporosis, accompanied by 12 million male subjects with high-risk conditions (Gielen et al., 2011). Cumulative data have suggested that osteoporosis-oriented fracture risk among male subjects is substantial, and the reports in the United States showed that 27–30% of fractures occur at ages of ≥ 50 years among male subjects (Burge et al., 2007; King et al., 2009). Worldwide, male subjects aged 50 years and older were estimated to incur 39% of all osteoporotic fractures (Johnell and Kanis, 2006). Also, the mortality rate after osteoporotic fracture in male subjects was found to be 39–52% higher than that in females (Haentjens et al., 2010; Kannegaard et al., 2010). Despite the abovementioned facts, osteoporosis in male subjects was still found to be undertreated or under-recognized, even though nearly one in five male subjects aged 50 years and older meet the criteria of the National Osteoporosis Foundation and other references to receive treatment with anti-osteoporosis therapies (Kiebzak et al., 2002; Ebeling, 2008; Khosla et al., 2008). With increasing longevity of male subjects and a concomitant increase in the proportion of the aging population, fractures and burdens for targeted subjects’ health care are likely to increase in the upcoming years.

Recently, the routine therapies for anti-osteoporosis in male subjects include alendronate, risedronate, and teriparatide (Watts et al., 2012; Viswanathan et al., 2018). Although a series of epidemiological reports have been conducted to assess the correlation between anti-osteoporosis medication and the potential risk of male subjects’ fractures (Ringe et al., 1998; Kurland et al., 2000; Orwoll et al., 2000; Ebeling et al., 2001; Ringe et al., 2001; Trovas et al., 2002; Orwoll et al., 2003; Ringe et al., 2004; Miller et al., 2004; Shimon et al., 2005; Toth et al., 2005; Ringe et al., 2006; Boonen et al., 2009; Ringe et al., 2009; Langdahl et al., 2009; Orwoll et al., 2010a; Orwoll et al., 2010b; Ringe et al., 2010; Boonen et al., 2011; Boonen et al., 2012; Orwoll et al., 2012; Kaufman et al., 2013; Kachnic et al., 2013; Nakamura et al., 2014; Yan, 2014; Peng et al., 2018; Zhao et al., 2018), the outcomes have remained controversial. An inverse link between anti-osteoporosis medication and the risk of male subjects’ fractures was observed in three studies (Orwoll et al., 2000; Ringe et al., 2006; Ringe et al., 2009), whereas no association was found in other studies (Ringe et al., 2001; Ringe et al., 2004; Boonen et al., 2009; Orwoll et al., 2010a; Orwoll et al., 2010b; Orwoll et al., 2012; Peng et al., 2018). The aim of this study was to evaluate the correlation between anti-osteoporosis medication and the risk of fracture in relation to low bone mass (including outcomes of osteoporosis, fracture, and BMD loss) in male subjects analyzed in studies within the updated literature.

## Methods

The guidelines reported in the Preferred Reporting Items for Systematic Reviews and Meta-analysis (PRISMA) (Moher et al., 2009) were strictly followed while performing the literature searches and during the assembling of the results. A protocol for this systematic review was developed before the research began; however, this review was not registered in the Research Registry.

### Data Sources and Search Strategies

Randomized controlled trials (RCTs) that analyzed the effectiveness of a treating prescription for male subjects with osteoporosis (or low BMD) and that focused on the outcomes of fracture were included. Relevant studies from Embase, PubMed, Web of Science, and Chinese database of CNKI were searched from the inception to January 30th, 2019. The strategies of free-text terms and MeSH terms were adopted for the databases with relevant key words such as “osteoporosis,” “osteopenia,” “fracture,” “bone density,” “bone mass,” “bone,” “bone disease,” “alendronate,” “risedronate,” “ibandronate,” “teriparatide,” and other routine anti-osteoporosis medications. Also, the search methodology included identification of MeSH words from the abstract and title. Additional articles were taken into consideration by manually rechecking the reference lists of topical review documents. The final results of the article searching were updated on January 30th, 2019. A full electronic search strategy for the PubMed database was included as an additional file (**Table S1**).

### Study Selection

Inclusion criteria were used for identification of the potential articles. The domains of inclusion criteria were as follows: 1) RCT studies published in Chinese or English, 2) studies focused on assessing the effectiveness of a treating prescription for subjects with low bone mass (including outcomes of osteoporosis, fracture, and BMD loss, e.g., age ≥ 18 years, males), 3) studies that provided separate results for male subjects or included male subjects, 4) studies that reported fracture outcomes or provided sufficient data to calculate numbers of male subjects involved, 5) the length of interventions in the studies was at least 6 months, and 6) studies had to address the trial and the control group (i.e., either comparison of intervention as anti-osteoporosis medication *versus* placebo, active comparators, or another group). Most of the included articles were taken in comparison to a treatment option with vitamin D and calcium, placebo, or both. Very few of the studies had comparative active compounds.

Furthermore, the exclusion criteria were as follows: 1) previous reports of review papers, mechanistic studies, or animal experiments; 2) the subjects were not all found to have low bone mineral density (BMD) (*T*-score ≤1) or osteoporosis; and 3) reports that published only abstracts. Articles were assessed for inclusion based on two stages. The first step consisted of carefully reviewing the titles and abstracts, while the second step consisted of full-text checking of the articles for indications related to the topics of our meta-analysis. Two staff members carried out the eligibility assessment and data extraction. The discrepancies were settled by consultation with another researcher.

### Extraction of Data and Quality Assessment

Data were collected from the articles and included the following items: the characteristics of the trial subjects, number of male subjects, study population, trial/control assessment, comparator for assessed trial/control, periods of the trial, the time of follow-ups, fracture-outcome domains, and results mentioned for fracture-outcome domains in the control and trial groups. As for the domain of fracture outcomes, we mainly gathered data on the numbers of subjects with incident fractures in the trial/control groups. Two researchers performed the above data extractions based on the standardized forms for literature collection. While literature was found and performed in the previous review, we would refer to the source document and recheck whether or not the information was correct. The data extraction was done independently and was further cross-checked for validity. Any discrepancies were resolved by seeking help from a third expert. The quality-assessment tool focused on the risk bias assessment recommended by the Cochrane Collaboration (RevMan 5.3; www.cochrane.org/training/cochrane-handbook). Key domains for the above assessments contained reporting bias, selection bias, detection bias, performance bias, attrition bias, and other sources of biases.

### Data Analysis

The clinical characteristics and study quality were performed by narrative synthesis. For the assessment of study quality, we used the approaches that are recommended by the Cochrane Collaboration to evaluate risk of potential bias for the studies included, including schemes to estimate the potential risk of performance, selection, attrition, detection, and reporting biases. Pooled relative risks (RRs) with 95% confidence intervals (CIs) were computed by using a fixed-effects model (FEM) if there was no significant evidence of heterogeneity. If this was not the case, we performed the evaluation using the random-effects model (REM). Between-trial heterogeneity in all the meta-analyses was calculated based on *I*-squared (*I* ^2^) statistics and Chi-square tests. For the articles that mentioned the outcomes of fracture for several time points of follow-up, the longest duration times were chosen while conducting meta-analyses. While the statistical heterogeneity was identified, we would conduct the sensitivity analyses to explore the potential sources for the existing heterogeneity. Publication biases were checked and inspected by funnel-plot asymmetry. The analyses above were carried out based on the software of Stata SE, version 14.1 (Stata Corp, College Station, TX, USA). RevMan 5.3 software, recommended by the Cochrane Collaboration, was used to assess study quality and potential sources of biases.

## Results

### Literature Search and Study Selection

**Figure 1:**
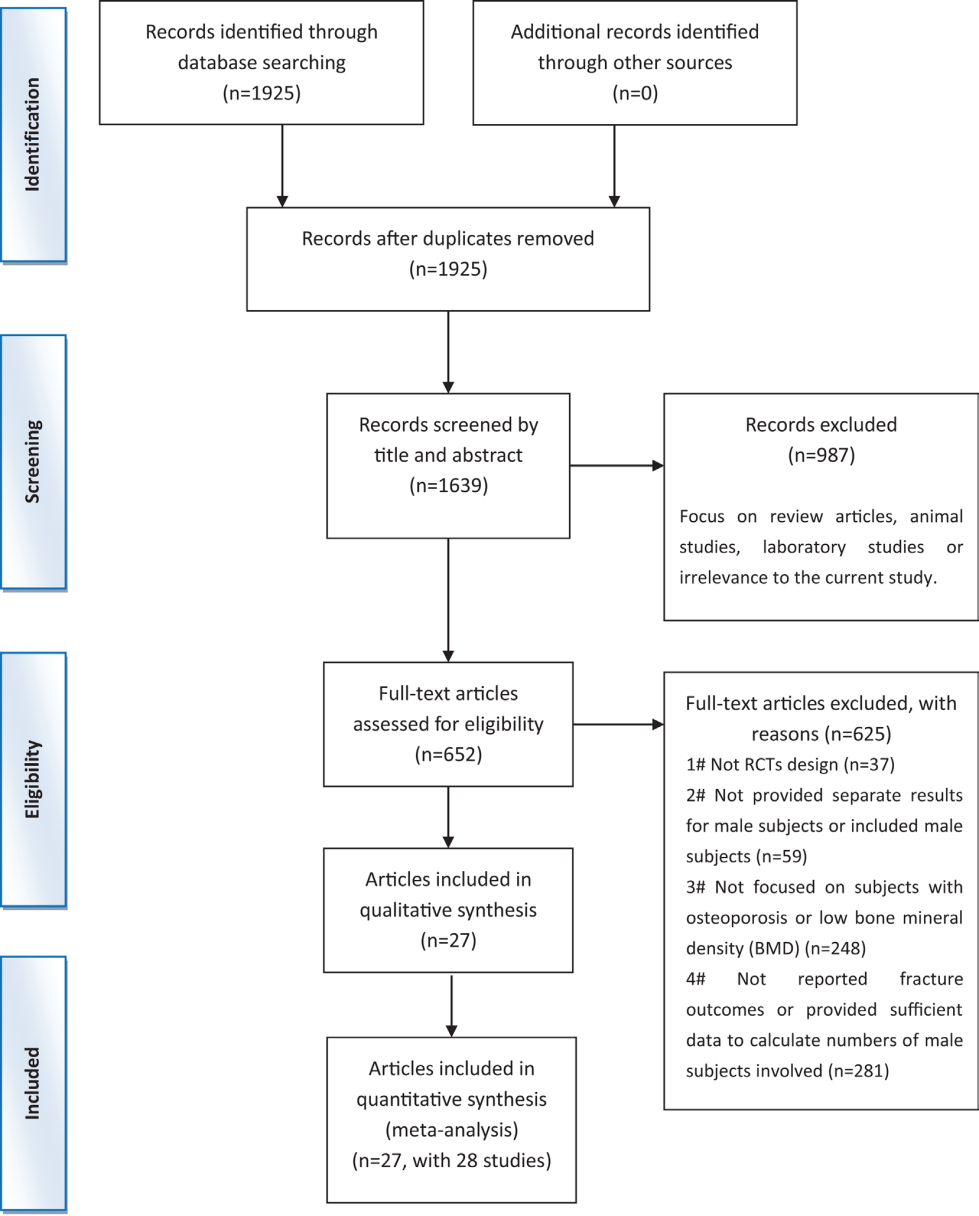
Preferred reporting items for systematic reviews and meta-analysis flow chart of the literature search.

Across the 1,639 documents identified during the initial search, 652 articles were selected for further verification by assessing the details of their full-text manuscripts. Then, 625 documents were excluded for various reasons (**Figure 1**). Finally, 27 articles (Ringe et al., 1998; Kurland et al., 2000; Orwoll et al., 2000; Ebeling et al., 2001; Ringe et al., 2001; Trovas et al., 2002; Orwoll et al., 2003; Miller et al., 2004; Ringe et al., 2004; Shimon et al., 2005; Toth et al., 2005; Ringe et al., 2006; Boonen et al., 2009; Langdahl et al., 2009; Ringe et al., 2009; Orwoll et al., 2010a; Orwoll et al., 2010b; Ringe et al., 2010; Boonen et al., 2011; Boonen et al., 2012; Orwoll et al., 2012; Kachnic et al., 2013; Kaufman et al., 2013; Nakamura et al., 2014; Yan, 2014; Peng et al., 2018; Zhao et al., 2018) (including 28 studies) met the qualified criteria. The procedure of the trial selection is presented in **Figure 1**.

### Study Characteristics

The characteristics of the 27 articles (Ringe et al., 1998; Kurland et al., 2000; Orwoll et al., 2000; Ebeling et al., 2001; Ringe et al., 2001; Trovas et al., 2002; Orwoll et al., 2003; Miller et al., 2004; Ringe et al., 2004; Shimon et al., 2005; Toth et al., 2005; Ringe et al., 2006; Boonen et al., 2009; Langdahl et al., 2009; Ringe et al., 2009; Orwoll et al., 2010a; Orwoll et al., 2010b; Ringe et al., 2010; Boonen et al., 2011; Boonen et al., 2012; Orwoll et al., 2012; Kachnic et al., 2013; Kaufman et al., 2013; Nakamura et al., 2014; Yan, 2014; Peng et al., 2018; Zhao et al., 2018) (including 28 studies) involving 5,678 subjects for this review are showed in **Table 1**. The studies were reported between 1998 and 2018. The durations of the included trials ranged from 6 to 36 months. The number of male subjects ranged from 24 to 1,199. The bisphosphonate prescriptions were found in the trial group across half of the studies included, and specified domains of alendronate or risedronate were used much more than the other bisphosphonates. Anti-osteoporosis treatment was compared with placebo, calcium, vitamin D, or combination of calcium and vitamin D for most of the studies, while active comparators were seldom observed in the control. Most of the fracture outcomes in the studies focused on vertebral fractures; also, studies contained the assessment of nonvertebral fractures or clinical fractures. The details are summarized in **Table 1**.

**Table 1:** Characteristics of the studies included and fracture outcomes.

First author, year (sources)	Study population	Subject description	Trial group	Control group	Number of subjects randomized (trial/control group)	Duration time	Number of subjects with incident fractures in trial group (n1/N1)	Number of men with incident fractures in control group (n2/N2)
Orwoll et al. (2000)	Multiple countries	Men with primary or hypogonadal osteoporosis. Age range 37–87 yrs^a^. Greater than 97% of participants were white.	Alendronate 10mg and Ca^b^ 500mg and vitamin D^c^ 400–450IU daily	Placebo and Ca^b^ 500mg and vitamin D^c^ 400–450IU daily	241 (146/95)	24 months	Vertebral fractures: 1/146; nonvertebral fractures: 6/146; clinical fractures: 7/146	Vertebral fractures: 7/95; nonvertebral fractures: 5/95; clinical fractures: 12/95
Ringe et al. (2001)	Germany	Men with primary osteoporosis, defined as a LS^d^ BMD T-score <–2.5	Alendronate 10mg and Ca^b^ 500mg daily	1-Alfacalcidol 1mcg and Ca^b^ 500mg daily	134 (68/66)	24 months	Vertebral fractures: 7/68; nonvertebral fractures: 6/68; clinical fractures: 13/68	Vertebral fractures: 16/66; nonvertebral fractures: 8/66; clinical fractures: 24/66
Ringe et al. (2004)	Germany	Men with primary osteoporosis, defined as a LS^d^ BMD T-score <–2.5	Alendronate 10mg and Ca^b^ 500mg daily	1-Alfacalcidol 1mcg and Ca^b^ 500mg daily	134 (68/66)	36 months	Vertebral fractures: 5/68; nonvertebral fractures: 6/68; clinical fractures: 11/68	Vertebral fractures: 12/66; nonvertebral fractures: 8/66; clinical fractures: 20/66
Miller et al. (2004)	United States	Men age 25–90 yrs^a^ with idiopathic or hypogonadal osteoporosis. Greater than 97% of participants were white.	Alendronic acid 70mg weekly + Ca^b^ 500mg and vitamin D^c^ 200IU twice daily	Placebo weekly + Ca^b^ 500mg and vitamin D^c^ 200IU twice daily	167 (109/58)	12 months	Vertebral fractures: 6/NP^h^ (7.5%); nonvertebral fractures: 6/NP^h^	Vertebral fractures: 3/NP^h^ (7.3%); nonvertebral fractures: 1/NP^h^
Shimon et al. (2005)	Israel	Men with hypogonadal osteoporosis with T-score <-2.0 at LS^d^ or FN^f^. Age range 29–69yrs^a^	Alendronate 10mg and Ca^b^ 800mg and vitamin D^c^ 600IU daily	Placebo and Ca^b^ 800mg and vitamin D^c^ 600IU daily	24 (11/13)	12 months	Vertebral fractures: 0/11; nonvertebral fractures: 0/11; clinical fractures: 0/11	Vertebral fractures: 0/13; nonvertebral fractures: 1/13; clinical fractures: 1/13
Peng et al. (2018)	China	Men with primary osteoporosis, defined as a LS^d^ BMD T-score <–2.5	Alendronate 10mg and Ca^b^ 800mg and vitamin D^c^ 600IU daily	Ca^b^ 800mg and vitamin D^c^ 600IU daily	80 (40/40)	6 months	Vertebral fractures: 1/40; nonvertebral fractures: 0/40; clinical fractures: 1/40	Vertebral fractures: 2/40; nonvertebral fractures: 3/40; clinical fractures: 5/40
Toth et al. (2005)	Hungary	Men with T-score at LS^d^ or FN^f^ <–2.5, no vertebral deformity, and no risk factors/signs of secondary osteoporosis. Age range 40–76 yrs^a^	Calcitonin 200IU nasal daily during alternate months + 1,000mg Ca^b^ and 400IU vitamin D^c^ daily	1,000mg Ca^b^ and 400IU vitamin D^c^ daily	71 (40/31)	18 months	Vertebral fractures: 0/40; nonvertebral fractures: 0/40; clinical fractures: 0/40	Vertebral fractures: 2/31; nonvertebral fractures: 1/31; clinical fractures: 3/31
Trovas et al. (2002)	Greece	Men with LS^d^ or FN^f^ BMD T-score <–2.5 and no secondary osteoporosis risk factors. Age range 27–74 yrs^a^	Salmon calcitonin (SCT) 200IU nasal + Ca^b^ 500mg daily	Placebo nasal + Ca^b^ 500mg daily	28 (15/13)	12 months	Vertebral fractures: 1/15; nonvertebral fractures: 0/15; clinical fractures: 1/15	Vertebral fractures: 2/13; nonvertebral fractures: 0/13; clinical fractures: 2/13
Nakamura et al. (2014)	Japan	Japanese subjects with osteoporosis age ≥ 50 yrs^a^ with 1–4 vertebral fractures (but not >2 moderate and/or any severe) and DXA T-score <–1.7 at LS^d^ or <–1.6 at TH^g^	Denosumab 60mg sq injection every 6 months + Ca^b^ 600mg and vitamin D^c^ 400IU daily	Placebo	47 (23/24)	24 months	Vertebral fractures: 0/23; nonvertebral fractures: 0/23; clinical fractures: 0/23	Vertebral fractures: 2/24; nonvertebral fractures: 0/24; clinical fractures: 2/24
Orwoll et al. (2012)	Multiple countries	Ambulatory men age 30–85 yrs^a^ with T-score ≤–2.0 and ≥–3.5 at the LS^d^ or FN^f^ or had prior major osteoporotic fracture and a T-score ≤–1.0 and ≥–3.5 at LS^d^ or FN^f^	Denosumab 60mg sq injection every 6 months + Ca^b^ ≥1,000mg and vitamin D^c^ ≥800IU daily	Placebo sq injection every 6 months + Ca^b^ ≥1,000mg and vitamin D^c^ ≥800IU daily	242 (121/121)	12 months	Vertebral fractures: 0/121; nonvertebral fractures: 1/121; clinical fractures: 1/121	Vertebral fractures: 1/121; nonvertebral fractures: 1/121; clinical fractures: 2/121
Boonen et al. (2009)	Multiple countries	Ambulatory men age ≥ 30 yrs^a^ with primary osteoporosis or hypogonadal osteoporosis who declined testosterone. Age range 36–83 yrs^a^. 95% were white.	Risedronate 35mg weekly + Ca^b^ 1,000mg and vitamin D^c^ 400–500IU daily	Ca^b^ 1,000mg and vitamin D^c^ 400–500IU daily	284 (191/93)	24 months	Vertebral fractures (24 months): 2/191; nonvertebral fractures (24 months): 7/191; clinical fractures (12 months, 24 months): 5/191, 9/191	Vertebral fractures (24 months): 0/93; nonvertebral fractures (24 months): 6/93; clinical fractures (12 months, 24 months): 3/93, 6/93
Ringe et al. (2009)	Germany	Men with primary or secondary osteoporosis, defined as LS^d^ BMD T-score ≤–2.5 and FN^f^ T-score ≤–2.0	Risedronate 5mg and Ca^b^ 1,000mg and vitamin D^c^ 800IU daily	Alfacalcidol 1mcg and Ca^b^ 500mg daily OR vitamin D^c^ 800–1,000IU and Ca^b^ 800–1,200mg daily	316 (158/158)	24 months	Vertebral fractures (12 months, 24 months): 8/158, 14/158; nonvertebral fractures (12 months, 24 months): 10/158, 18/158; clinical fractures (12 months, 24 months): 18/158, 32/158	Vertebral fractures(12 months, 24 months): 20/158, 35/158; nonvertebral fractures (12 months, 24 months): 17/158, 33/158; clinical fractures (12 months, 24 months): 37/158, 68/158
Ringe et al. (2006)	Germany	Men with primary or secondary osteoporosis, defined as LS^d^ BMD T-score ≤–2.5 and FN^f^ T-score ≤–2.0	Risedronate 5mg and Ca^b^ 1,000mg and vitamin D^c^ 800IU daily	Alfacalcidol 1mcg and Ca^b^ 500mg daily OR vitamin D^c^ 800–1,000IU and Ca^b^ 800–1,200mg daily	316 (158/158)	12 months	Vertebral fractures: 8/158; nonvertebral fractures: 10/158; clinical fractures: 18/158	Vertebral fractures: 20/158; nonvertebral fractures: 17/158; clinical fractures: 37/158
Boonen et al. (2011)	Multiple countries	Men within 90 days of surgical repair of a low-trauma hip fracture who were ambulatory without assistive device before fracture and unwilling/unable to take oral bisphosphonate. 93.5% Caucasian	Zoledronic acid 5mg IV yearly + loading dose of vitamin D^c^ + Ca^b^ 1,000–1,500mg and vitamin D^c^ 400–800IU daily	Placebo IV infusion yearly + loading dose of vitamin D^c^ + Ca^b^ 1,000–1,500mg and vitamin D^c^ 400–800IU daily	508 (248/260)	36 months; median 1.9 years	Clinical fracture (excluding facial/digital/pathological fractures) at 24 months: 16/248	Clinical fracture (excluding facial/digital/pathological fractures) at 24 months: 20/260
Boonen et al. (2012)	Multiple countries	Men with primary or hypogonadism-associated osteoporosis who were 50–85 yrs^a^ of age	Zoledronic acid IV yearly + Ca^b^ 1,000– 1,500mg and vitamin D^c^ 800–1,200IU daily	Placebo IV infusion yearly + Ca^b^ 1,000– 1,500mg and vitamin D^c^ 800–1,200IU daily	1,199 (588/611)	24 months	Vertebral fractures: 1/588; nonvertebral fractures: 5/588; clinical fractures (vertebral and nonvertebral): 6/588	Vertebral fractures: 3/611; nonvertebral fractures: 8/611; clinical fractures (vertebral and nonvertebral): 11/611
Ebeling et al. (2001)	Australia	Caucasian men with primary osteoporosis age 27–77 yrs^a^ w/ ≥1 fragility fracture	Calcitriol 0.25mcg and placebo Ca^b^ tablets twice daily	Ca^b^ 500mg and placebo calcitriol capsules twice daily	41 (20/19 evaluable for analysis)	24 months	Vertebral fractures (12 months, 24 months): 3/20, 6/20; nonvertebral fractures (24 months): 5/20; clinical fractures: 11/20	Vertebral fractures (12 months, 24 months): 1/19, 1/19; nonvertebral fractures (24 months): 0/19; clinical fractures: 1 /19
Orwoll et al. (2010a)	United States	Men age ≥ 30 yrs^a^ with baseline FN^f^ T-scores ≤–2.0 and LS^d^ T-scores ≤–1.0 or LS^d^ T-scores ≤–2.0, FN^f^ T-scores ≤–1.0, and T-scores ≥–4.0 at any site; and no vertebral fractures 95% were white.	Ibandronate 150mg oral monthly + Ca^b^ 1,000mg and vitamin D^c^ 400IU daily	Placebo oral monthly + Ca^b^ 1,000mg and vitamin D^c^ 400IU daily	135 (87/48)	12 months	Vertebral fractures: 1/87; nonvertebral fractures: 2/87; clinical fractures (vertebral and nonvertebral): 3/87	Vertebral fractures: 2/48; nonvertebral fractures: 0/48; clinical fractures (vertebral and nonvertebral): 2/48
Orwoll et al. (2010b)	Multiple countries	Men age 25–85 yrs^a^ with primary or hypogonadal osteoporosis, a BMD T-score of –2.0 at FN^f^ and –1.0 at LS^d^ or –1.0 at FN^f^ with prior low-trauma fracture or radiographic vertebral fracture. ≈95% Caucasian.	Zoledronic acid 5mg IV yearly + oral placebo capsule weekly + Ca^b^ 1,000mg and vitamin D^c^ 800–1,000IU daily	Alendronate 70mg oral capsule weekly + placebo IV infusion yearly + Ca^b^ 1,000mg and vitamin D^c^ 800–1,000IU daily	302 (154/148)	24 months	Vertebral fractures: 4/154; nonvertebral fractures: 0/154; clinical fractures: 4/154	Vertebral fractures: 6/148; nonvertebral fractures: 0/148; clinical fractures: 6/148
Ringe et al. (1998)	Germany	Men with T-score at LS^d^ <–2.5, no x-ray significant deformity or signs of prior vertebral fractures, and no significant risk factors for/signs of secondary osteoporosis. Age range 33–68 yrs^a^	Monofluorophosphate (MFP) 114mg daily (3 months on, 1 month off) + Ca^b^ 950–1,000mg daily	Ca^b^ 1,000mg daily	64 (32/32)	36 months	Vertebral fractures: 3/32; nonvertebral fractures: 5/32; clinical fractures: 8/32	Vertebral fractures: 12/32; nonvertebral fractures: 8/32; clinical fractures: 20/32
Kurland et al. (2000)	United States	Men with idiopathic osteoporosis age 30–68 yrs^a^	PTH-(1–34) (Teriparatide) 400IU sq injection and Ca^b^ 1,500mg and vitamin D^c^ 400IU daily	Placebo sq injection and Ca^b^ 1,500mg and vitamin D^c^ 400IU daily	23 (10/13)	18 months	Vertebral fractures (after 1 year of treatment): 1/10; nonvertebral fractures: 0/10; clinical fractures: 1/10	Vertebral fractures (after 1 year of treatment): 2/13; nonvertebral fractures: 0/13; clinical fractures: 2/13
Zhao et al. (2018)	China	Men age 30–70 yrs^a^ with T-score ≤−2.5 at the LS^d^, FN^f^ or TH^g^	PTH-(1–34) (teriparatide) 400IU sq injection and vitamin D^c^ 400IU daily	Ca^b^ 1,500mg and vitamin D^c^ 400IU daily	88 (44/44)	12 months	Vertebral fractures: 1/44; nonvertebral fractures: 3/44; clinical fractures: 4/44	Vertebral fractures: 2/44; nonvertebral fractures: 6/44; clinical fractures: 8/44
Kaufman et al. (2013)	Multiple countries	Ambulatory white men age ≥ 65 yrs^a^ with low BMD (LS^d^ T-score ≤–2.5 and/or FN^f^ T-score ≤–2.4) and ≥1 risk factor for osteoporotic fracture	Strontium ranelate 2g and Ca^b^ 1,000mg and vitamin D^c^ 800IU daily	Placebo oral and Ca^b^ 1,000mg and vitamin D^c^ 800IU daily	261 (174/87)	24 months	Vertebral fractures: 7/174; nonvertebral fractures: 0/174; clinical fractures: 7/174	Vertebral fractures: 5/87; nonvertebral fractures: 0/87; clinical fractures: 5/87
Ringe et al. (2010)	Germany	Men age 40–75 yrs^a^ with primary osteoporosis (LS^d^ T-score <–2.5 and ≥1 prevalent vertebral fracture) with a LS^d^ T-score <–3 and T-score at TH^g^ <–2.	Strontium ranelate 2g and Ca^b^ 1,200mg and vitamin D^c^ 800IU daily	Alendronate 70mg weekly + Ca^b^ 1,200mg and vitamin D^c^ 800IU daily	152 (76/76)	12 months	Vertebral fractures: 4/76; nonvertebral fractures: 3/76; clinical fractures: 7/176	Vertebral fractures: 4/76; nonvertebral fractures: 6/76; clinical fractures: 10/76
Yan (2014)	China	Men with primary osteoporosis who were ≥60 yrs^a^ of age	Strontium ranelate 2g and vitamin D^c^ 800IU daily	Ca^b^ 1,000mg and vitamin D^c^ 800IU daily	58 (26/32)	12 months	Vertebral fractures: 1/26; nonvertebral fractures: 0/26; clinical fractures: 1/26	Vertebral fractures: 2/32; nonvertebral fractures: 0/32; clinical fractures: 2/32
Langdahl et al. (2009)	Multiple countries	Patients with glucocorticoid-induced osteoporosis	Teriparatide 20mcg sq injection and oral placebo and Ca^b^ 1,000mg and vitamin D^c^ 800IU daily	Alendronate 10mg oral and placebo sq injection and Ca^b^ 1,000mg and vitamin D^c^ 800IU daily	83 (42/41)	18 months	Vertebral fractures: 0/42; nonvertebral fractures: 1/42; clinical fractures: 1/42	Vertebral fractures: 4/41; nonvertebral fractures: 2/41; clinical fractures: 6/41
Orwoll et al. (2003) (with teriparatide 20 µg)	Multiple countries	Ambulatory men age 30–85 yrs^a^ with idiopathic or hypogonadal osteoporosis with LS^d^ or proximal femur BMD at least 2SD below young adult mean for men, free of other chronic, disabling conditions. 99% were white.	Teriparatide 20mcg sq self-injection and Ca^b^ 1,000mg and vitamin D^c^ 400–1,200IU daily	Placebo sq self-injection and Ca^b^ 1,000mg and vitamin D^c^ 400–1,200IU daily	298 (151 teriparatide 20 µg /147 placebo)	24 months planned; median treatment was 11 months (range 2–15)	Vertebral fractures: 0/151; nonvertebral fractures: 2/151; clinical fractures: 2/151	Vertebral fractures: 0/147; nonvertebral fractures: 3/147; clinical fractures: 3/147
Orwoll et al. (2003)(with teriparatide 20 µg)	Multiple countries	Ambulatory men age 30–85 yrs^a^ with idiopathic or hypogonadal osteoporosis with LS^d^ or proximal femur BMD at least 2SD below young adult mean for men, free of other chronic, disabling conditions. 99% were white.	Teriparatide 40mcg sq self-injection and Ca^b^ 1,000mg and vitamin D^c^ 400–1,200IU daily	Placebo sq self-injection and Ca^b^ 1,000mg and vitamin D^c^ 400–1,200IU daily	286(139 teriparatide 40 µg /147 placebo)	24 months planned; median treatment was 11 months (range 2–15)	Vertebral fractures: 0/139; nonvertebral fractures: 1/139; clinical fractures: 1/139	Vertebral fractures: 0/147; nonvertebral fractures: 3/147; clinical fractures: 3/147
Kachnic et al. (2013)	United States	Men receiving LHRH and RT for locally advanced prostate adenocarcinoma with low BMD (but not osteoporosis) and negative bone scans. Age range 51–87 yrs^a^. 93% were white.	Zoledronic acid 4mg IV every 6 months + Ca^b^ 500mg and vitamin D^c^ 400IU daily	Ca^b^ 500mg and vitamin D^c^ 400IU daily	96 (50/46)	36 months	Clinical fractures (any bone fracture): 1/50	Clinical fractures (any bone fracture): 1/46

Annotation: a, years; b, calcium; c, vitamin D; d, lumbar spine; f, femoral neck; g, total hip; h, not provided or completed from reported data.

### Study Quality and Potential Sources of Bias

The risks of bias for the 27 articles (Ringe et al., 1998; Kurland et al., 2000; Orwoll et al., 2000; Ebeling et al., 2001; Ringe et al., 2001; Trovas et al., 2002; Orwoll et al., 2003; Miller et al., 2004; Ringe et al., 2004; Shimon et al., 2005; Toth et al., 2005; Ringe et al., 2006; Boonen et al., 2009; Langdahl et al., 2009; Ringe et al., 2009; Orwoll et al., 2010a; Orwoll et al., 2010b; Ringe et al., 2010; Boonen et al., 2011; Boonen et al., 2012; Orwoll et al., 2012; Kachnic et al., 2013; Kaufman et al., 2013; Nakamura et al., 2014; Yan, 2014; Peng et al., 2018; Zhao et al., 2018) (including 28 studies) are presented in **Figures S1**, **S2**. The reporting quality of the included trials was identified to be generally moderate, which provided certain unclear information (i.e., whether concealment of allocation, the random sequence generated, and items of blinding were carried out) for further verified inference. Additionally, inadequate reporting could result in risk validity of the results or potential bias.

### Bisphosphonate and the Risk of Osteoporotic Fractures

**Figure 2:**
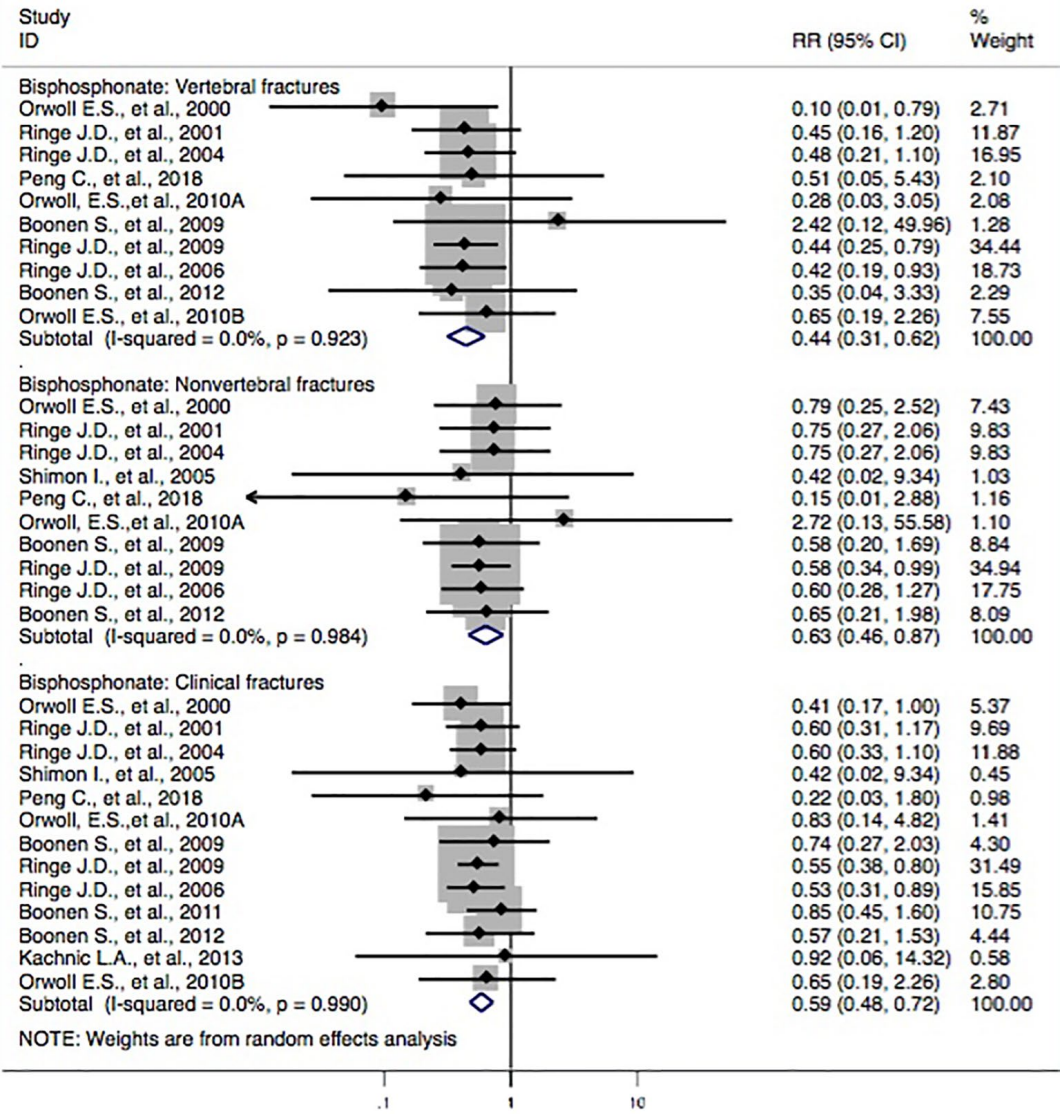
Forest plot of meta-analysis on bisphosphonate and osteoporotic fractures. The size of the diamond and box is positively proportional to the weight assigned to each study, and horizontal lines represent the 95% CI. RR, relative risk; CI, confidence interval.

Thirteen studies (Orwoll et al., 2000; Ringe et al., 2001; Ringe et al., 2004; Ringe et al., 2006; Boonen et al., 2009; Ringe et al., 2009; Orwoll et al., 2010a; Orwoll et al., 2010b; Orwoll et al., 2012; Peng et al., 2018) reported bisphosphonate treatments and the incidence of vertebral fractures. Three studies (Orwoll et al., 2000; Ringe et al., 2006; Ringe et al., 2009) revealed that the subjects with bisphosphonate probably reduced the risk of vertebral fractures, whereas the others (Ringe et al., 2001; Ringe et al., 2004; Boonen et al., 2009; Orwoll et al., 2010a; Orwoll et al., 2010b; Orwoll et al., 2012; Peng et al., 2018) failed to find such a correlation. The pooled-effect estimates showed that statistically significant differences between the two groups (RR, 0.44 [95% CI, 0.31–0.62]) were observed. Moreover, 10 studies (Orwoll et al., 2000; Ebeling et al., 2001; Ringe et al., 2001; Shimon et al., 2005; Ringe et al., 2006; Boonen et al., 2009; Ringe et al., 2009; Orwoll et al., 2010a; Boonen et al., 2012; Peng et al., 2018) reported the correlation between bisphosphonate and the incidence of nonvertebral fractures. In comparison to the control, a statistically significant association between the two groups (RR, 0.63 [95% CI, 0.46–0.87]) was identified. In addition, two studies (Boonen et al., 2009; Ringe et al., 2009) found that the subjects with bisphosphonate had a lower risk of clinical fractures, whereas the others (Orwoll et al., 2000; Ringe et al., 2001; Ringe et al., 2004; Shimon et al., 2005; Ringe et al., 2006; Orwoll et al., 2010a; Orwoll et al., 2010b; Boonen et al., 2011; Boonen et al., 2012; Kachnic et al., 2013; Peng et al., 2018) failed to show such a link. The synthesized evidence for the risk of clinical fractures displayed that there were statistically significant differences between groups (RR, 0.59 [95% CI, 0.48–0.72]). Overall, the results indicated that the patients with bisphosphonate were seemingly associated with lower risk of fracture outcomes, including vertebral fractures, nonvertebral fractures, and clinical fractures (**Figure 2**).

### Alendronate and the Risk of Osteoporotic Fractures

**Figure 3:**
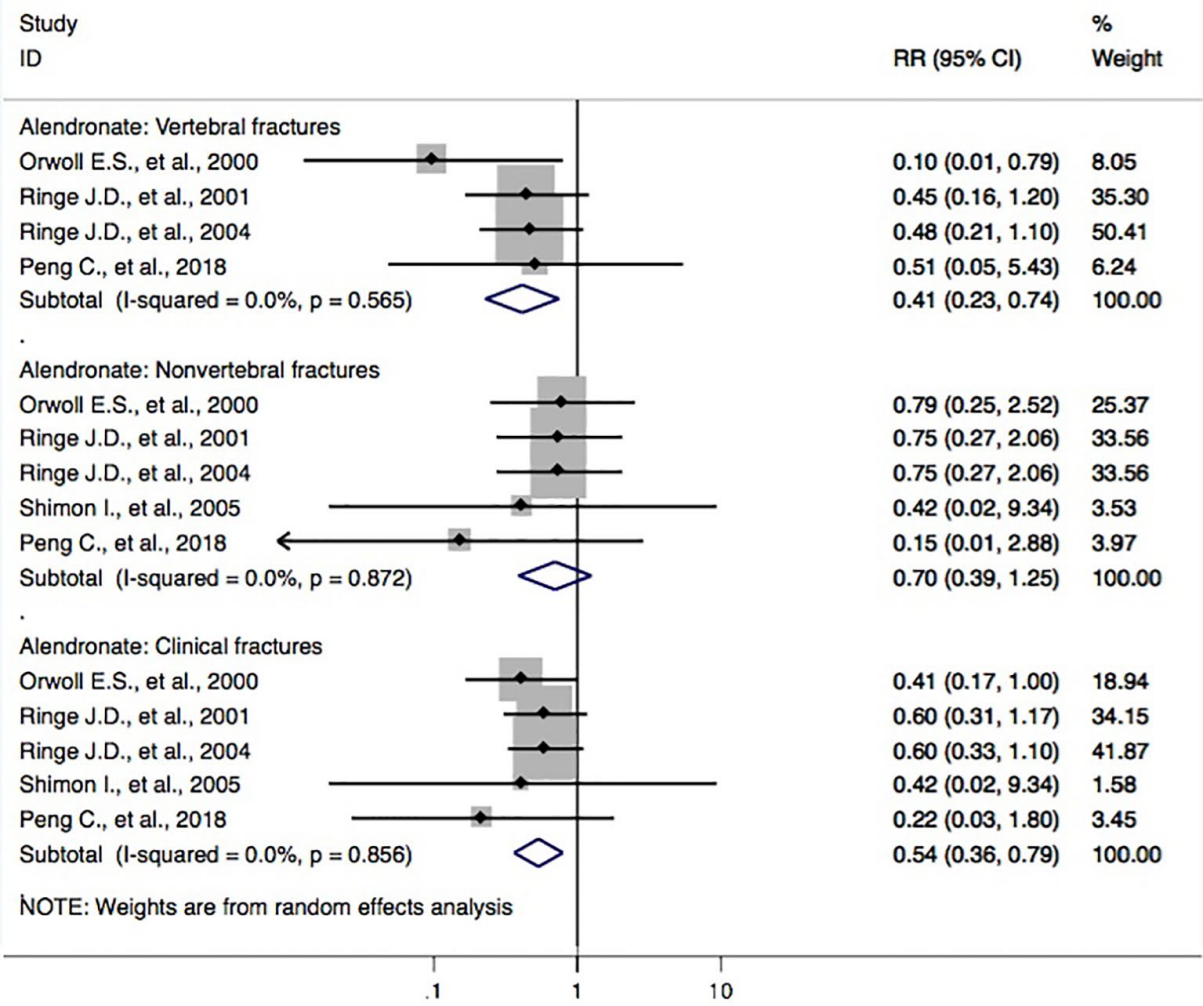
Forest plot of meta-analysis on alendronate and osteoporotic fractures. The size of diamond and box is positively proportional to the weight assigned to each study, and horizontal lines represent the 95% CI. RR, relative risk; CI, confidence interval.

Five studies (Orwoll et al., 2000; Ringe et al., 2001; Ringe et al., 2004; Shimon et al., 2005; Peng et al., 2018) reported alendronate and the incidence of osteoporotic fractures. The RR for the risk of vertebral fractures between groups was found (RR, 0.41 [95% CI, 0.23–0.74]). The outcomes showed that patients with alendronate had a 59% (95% CI, 26%–77%) lower risk of vertebral fractures. Also, the pooled-effect estimates for subjects with alendronate were toward a reduced risk of clinical fractures (RR, 0.54 [95% CI, 0.36–0.79]); however, this was not the case for nonvertebral fractures (RR, 0.70 [95% CI, 0.39–1.25]) when compared to that of the controls (**Figure 3**).

### Calcitonin and the Risk of Osteoporotic Fractures

There were two studies (Trovas et al., 2002; Toth et al., 2005) involving calcitonin and the incidence of osteoporotic fractures. Compared with that of the control, no statistically significant association was observed in the pooled analysis for calcitonin and the risk of the vertebral fracture domain (RR, 0.32 [95% CI, 0.05–1.98]), nonvertebral fracture domain (RR, 0.27 [95% CI, 0.01–6.37]), or clinical fracture domain (RR, 0.28 [95% CI, 0.05–1.72]) (**Figure S3**).

### Denosumab and the Risk of Osteoporotic Fractures

Two studies (Orwoll et al., 2012; Nakamura et al., 2014) evaluated denosumab and the incidence of osteoporotic fractures. The meta-analysis revealed that no statistically significant differences were found between groups concerning the risk of the vertebral fracture domain (RR, 0.27 [95% CI, 0.03–2.40]), nonvertebral fracture domain (RR, 1.00 [95% CI, 0.06–15.81]), or clinical fracture domain (RR, 0.37 [95% CI, 0.06–2.38]) (**Figure S4**).

### Risedronate and the Risk of Osteoporotic Fractures

**Figure 4:**
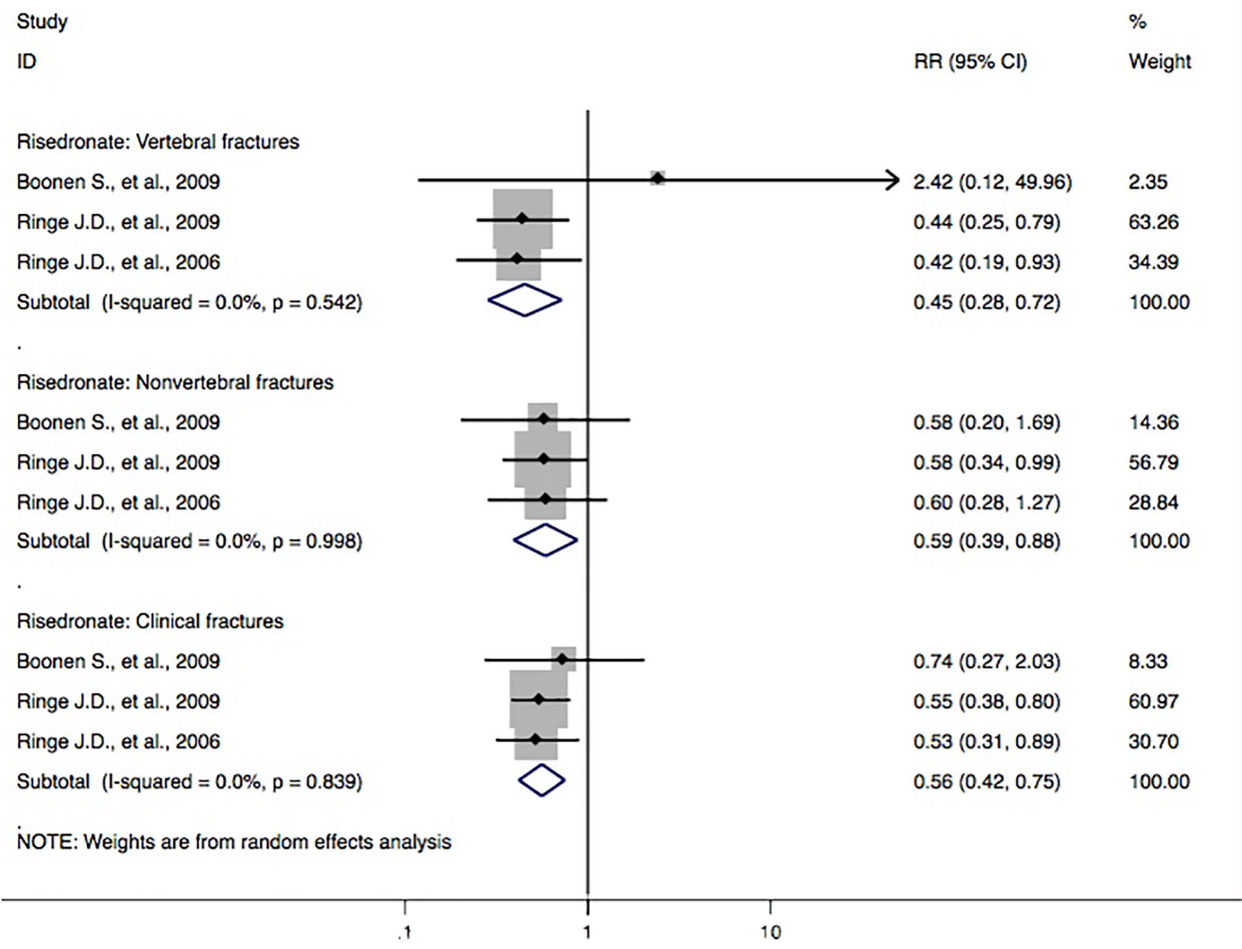
Forest plot of meta-analysis on risedronate and osteoporotic fractures. The size of diamond and box is positively proportional to the weight assigned to each study, and horizontal lines represent the 95% CI. RR, relative risk; CI, confidence interval.

Three studies (Ringe et al., 2006; Boonen et al., 2009; Ringe et al., 2009) reported risedronate and the incidence of vertebral fractures. Meta-analyses from the above trials found a significant reduction in fracture outcomes *via* administration of risedronate, including the risk of the vertebral fracture domain (RR, 0.45 [95% CI, 0.28–0.72]), nonvertebral fracture domain (RR, 0.59 [95% CI, 0.39–0.88]), and clinical fracture domain (RR, 0.56 [95% CI, 0.42–0.75]) (**Figure 4**).

### Calcitriol and the Risk of Osteoporotic Fractures

No statistically significant association was observed between calcitriol and risk of the vertebral fracture domain (RR, 4.62 [95% CI, 0.60–35.32]), nonvertebral fracture domain (RR, 8.46 [95% CI, 0.50–144.21]), or clinical fracture domain (RR, 7.10 [95% CI, 0.99–50.81]) (**Figure S5**).

### Ibandronate and the Risk of Osteoporotic Fractures

For the risk of osteoporotic fractures, no significant effect was identified in the ibandronate group in terms of the vertebral fracture domain (RR, 0.28 [95% CI, 0.03–3.05]), nonvertebral fracture domain (RR, 2.72 [95% CI, 0.13–55.58]), or clinical fracture domain (RR, 0.83 [95% CI, 0.14–4.82]) (**Figure S6**).

### Monofluorophosphate and the Risk of Osteoporotic Fractures

No positive effects were observed between monofluorophosphate and the risk of the vertebral fracture domain (RR, 0.31 [95% CI, 0.10–1.03]), nonvertebral fracture domain (RR, 0.68 [95% CI, 0.24–1.88]), or clinical fracture domain (RR, 0.52 [95% CI, 0.26–1.06]) (**Figure S7**).

### Strontium Ranelate and the Risk of Osteoporotic Fractures

Three studies (Ringe et al., 2010; Kaufman et al., 2013; Yan, 2014) reported strontium ranelate and the incidence of osteoporotic fractures. The overall effects of pooled analyses did not indicate any significant difference between groups concerning the risk of the vertebral fracture domain (RR, 0.79 [95% CI, 0.35–1.78]), nonvertebral fracture domain (RR, 0.52 [95% CI, 0.13–2.00]), or clinical fracture domain (RR, 0.71 [95% CI, 0.36–1.40]) (**Figure S8**).

### Teriparatide and the Risk of Osteoporotic Fractures

Five studies (Kurland et al., 2000; Orwoll et al., 2003; Langdahl et al., 2009; Zhao et al., 2018) (two studies included in Orwoll et al., 2003) evaluated teriparatide and the incidence of osteoporotic fractures. Meta-analysis of the data found that the subjects treated with teriparatide in the trial group were not significantly improved compared to those of the control concerning the reduction of fracture outcome, including the vertebral fracture domain (RR, 0.40 [95% CI, 0.10–1.67]), nonvertebral fracture domain (RR, 0.52 [95% CI, 0.21–1.27]), and clinical fracture domain (RR, 0.47 [95% CI, 0.22–1.02]) (**Figure S9**).

### Zoledronic Acid and the Risk of Osteoporotic Fractures

Four studies (Orwoll et al., 2010b; Boonen et al., 2011; Boonen et al., 2012; Kachnic et al., 2013) reported zoledronic acid and the incidence of osteoporotic fractures. As shown in **Figure S10**, no statistically significant differences between groups were identified for pooled effects by assessing the vertebral fracture domain (RR, 0.56 [95% CI, 0.19–1.67]), nonvertebral fracture domain (RR, 0.65 [95% CI, 0.21–1.98]), and clinical fracture domain (RR, 0.74 [95% CI, 0.46–1.21]).

### Publication Bias

Publication bias of this study was evaluated based on funnel plots and RRs performed from trials involving alendronate, risedronate, bisphosphonates, and the risk of fracture outcomes. To large extents, the points should be displayed symmetrically around the vertical line concerning the pooled RRs while in the absence of publication bias. The shapes of the funnel plot were found to be symmetrically reasonable, which indicated the absence of publication bias (**Figures S11**–**13**).

## Discussion

### Statement of Principal Findings

This review included 27 documents (involving 28 studies) with 5,678 subjects. Meta-analyses of these studies found that for the category of bisphosphonates, significant results were observed in pooled analyses for reduced risk of the vertebral fracture domain (RR, 0.44 [95% CI, 0.31–0.62]), nonvertebral fracture domain (RR, 0.63 [95% CI, 0.46–0.87]), and clinical fracture domain (RR, 0.59 [95% CI, 0.48–0.72]), compared with those of controls. Participants with bisphosphonates had a 56% (95% CI = 38–69%) lower risk of vertebral fractures, 37% (95% CI = 13–54%) lower risk of nonvertebral fractures, and 41% (95% CI = 28–52%) lower risk of clinical fractures. Furthermore, meta-analyses showed a decreased risk of vertebral fractures by treatment with alendronate and risedronate, but not with calcitonin, denosumab, calcitriol, ibandronate, monofluorophosphate, strontium ranelate, teriparatide, or zoledronic acid, as compared to that of the controls.

This systematic review confirms that bisphosphonates were correlated with a lower risk of the vertebral fracture domain, nonvertebral fracture domain, and clinical fracture domain for male subjects with osteoporosis. Because of the existing defects in the methodological quality for the included studies, the definitive correlation involving routine anti-osteoporosis medication and the risk of male subjects’ fracture could not be fully verified by the current evidence. Thus, any recent advice and proposals for clinical practice should be interpreted with caution. Further studies with high-quality designs are needed to validate these findings.

### Comparison of Findings With Other Results in the Literature

In this study, we performed a systematic review of the possible protective effect of routine anti-osteoporosis medication on the risk of fractures in male subjects with osteoporosis. A similar article (Nayak and Greenspan, 2017) with 22 studies included on the same subjects had been reported in March 2017. This study found that bisphosphonates as a treatment category significantly lowered the risk of the vertebral fracture domain (RR, 0.368 [95% CI, 0.252–0.537]) and nonvertebral fracture domain (RR, 0.604 [95% CI, 0.404–0.904]) compared to that of controls. In our study, three more studies (Yan, 2014; Peng et al., 2018; Zhao et al., 2018), conducted in China, were included. Three studies (Orwoll et al., 2003; Miller et al., 2004; Shimon et al., 2005) in the previous article (Nayak and Greenspan, 2017) were not included in their meta-analysis, but we rechecked these studies and chose to include them for further analysis. To some extent, our present study provides the opportunity to evaluate the original documents that have been reported in Chinese journals. This also contributes to the possible benefit of assessing published articles and forming a sound basis for further research for similar topics. In addition to assessing the correlation between anti-osteoporosis medications and the risk of fractures, we also performed the meta-analyses on the possibly beneficial effect of bisphosphonate and the risk of fracture in relation to low bone mass (including outcomes of osteoporosis, fracture, and BMD loss) in male subjects. Finally, we found that the pooled-effect estimates of bisphosphonates were observed in the direction of lower risk of the vertebral fracture domain (RR, 0.44 [95% CI, 0.31–0.62]), nonvertebral fracture domain (RR, 0.63 [95% CI, 0.46–0.87]), and clinical fracture domain (RR, 0.59 [95% CI, 0.48–0.72]), compared with those of the controls. Participants with bisphosphonates had a 56% (95% CI = 38–69%) lower risk of vertebral fractures, 37% (95% CI = 13–54%) lower risk of nonvertebral fractures, and 41% (95% CI = 28–52%) lower risk of clinical fractures (**Figure 2**). These findings were different from the results of Nayak et al. that were published in March 2017 (Nayak and Greenspan, 2017). Furthermore, our meta-analyses also showed a decreased risk of the vertebral fracture domain by treatment with alendronate (RR, 0.41 [95% CI, 0.23–0.74]; **Figure 3**) and risedronate (RR, 0.45 [95% CI, 0.28–0.72]; **Figure 4**), compared with that of controls; however, this association was not found among calcitonin, denosumab, calcitriol, ibandronate, monofluorophosphate, strontium ranelate, teriparatide, or zoledronic acid (**Figures S3**–**10**).

A number of articles concerning anti-osteoporosis medication and possible risk of fracture have been published and have included randomized controlled studies, case series, controlled studies, case reports, and meta-analyses. However, there was no systematic review focusing upon possible protective effects of bisphosphonates and other routine anti-osteoporosis medications on the risk of fracture of osteoporosis male subjects in relation to low bone mass (including outcomes of osteoporosis, fracture, and BMD loss). To a certain extent, our study is the first to explore the roles of anti-osteoporosis medication and possible risk of the above outcome measures by searching and analyzing the updated evidence of the existing literature.

### Possible Explanations and Implications of the Study

Additive benefits for the subjects with male fracture in relation to low bone mass were observed while taking bisphosphonates (i.e., a significant links possibly exist among bisphosphonates, fracture, osteoporosis, and BMD loss in male subjects). Relatively few RCTs have been carried out to evaluate the effectiveness of anti-osteoporosis therapies for male subjects concerning risk reduction of fracture outcomes. These systematic-review findings for individual treatment prescriptions indicated that risedronate and alendronate could decrease the risk of the vertebral fracture domain of male subjects. However, the review for other specified treatment prescriptions did not reveal evidence of sufficient efficacy for reducing the risk of the vertebral fracture domain for male subjects, including treatments with denosumab, calcitriol, calcitonin, ibandronate, monofluorophosphate, strontium ranelate, teriparatide, and zoledronic acid. For the category of bisphosphonates, significant results were observed in pooled analyses for lower risk of vertebral fractures, nonvertebral fractures, and clinical fractures when compared to those of the controls.

These findings highlight the need for additional RCTs with higher quality and better design to focus on the effectiveness of anti-osteoporosis therapies for male subjects that are fully efficacious for improving fracture outcomes. Moreover, these findings call for further studies to assess the effectiveness of non-bisphosphonate prescription options. Also, this review highlights the lack of active comparator RCTs of anti-osteoporosis therapies for male subjects. Additional studies of anti-osteoporosis therapies for male subjects using active comparators (not placebo) may contribute to further verifying the potential effectiveness of varied treatment prescriptions for decreasing the risk of fracture in male subjects. Furthermore, these findings indicate the demand for a larger diversity of subjects in actual clinical studies of anti-osteoporosis therapies for male subjects, because most of the articles greatly enrolled a trial population of white subjects. Finally, no included articles were observed for a relatively longer duration (≥3 years). Therefore, the effectiveness of relatively longer anti-osteoporosis periods to lower the risk of fracture for male subjects remains uncertain. Further studies with longer periods and follow-up times would be helpful for assessing the effectiveness of anti-osteoporosis therapies for more than 3 years on the risk of fracture in male subjects. This process maybe help to clarify the possible fracture-risk reduction benefit for male subjects, similar to those of longer duration anti-osteoporosis studies that have shown the positive effects for females (Black et al., 2012; Cosman et al., 2014).

### Strengths of the Review

Several notable strengths have been observed in this systematic review. First and foremost, this study is the most comprehensive systematic review of RCTs concerning effectiveness of potential anti-osteoporosis medication to lower the risk of male subjects’ fractures in relation to low bone mass (including outcomes of osteoporosis, fracture, and BMD loss). Despite a similar article (Nayak and Greenspan, 2017) with 22 studies reported in March 2017, another previous review on a similar topic of osteoporosis treatment efficacy for male subjects was also published in 2011; however, this study only included five articles that reported outcomes as fracture and concluded that the studies were inconclusive in terms of any decrease of fracture risk in male subjects (Schwarz et al., 2011). Our systematic review included 27 RCT articles (with 28 studies) involving anti-osteoporosis therapies for male subjects that reported outcomes of fractures and identified evidence involving the effectiveness of bisphosphonate prescriptions on lowering the risk of the vertebral fracture domain, nonvertebral fracture domain, and clinical fracture domain in male subjects. Furthermore, the risk of bias concerning individual RCTs included in this systematic review was assessed and performed based on the routine criteria that the Cochrane Collaboration recommended (Higgins et al., 2011).

### Limitations of the Review

The meta-analyses were limited by the number of similar articles evaluating each individual treatment prescription, with just a few (ranging from one to six) articles including individual meta-analysis of the conducted treatment prescription. Also, meta-analyses with higher quality and better designs should be carried out in the future for investigating different fracture outcomes while assessing the category of bisphosphonates. Moreover, most articles included in this review had a relatively small sample size. The quality of the included studies in this systematic review was moderate. In addition, the findings of our meta-analysis were relatively limited due to the unclear or high risk of bias observed among the studies. Despite the above limitations, this review recommends that bisphosphonates could be adopted as first-line anti-osteoporosis therapies for male subjects, based on the evidence of their effectiveness in lowering the risk of vertebral fractures, nonvertebral fractures, and clinical fractures. The findings of the specified effectiveness concerning bisphosphonate in lowering the risk of fracture in male subjects apply to those individuals with low BMD or osteoporosis based on dual X-ray absorptiometry (DXA) criteria, because the articles included in this review enrolled subjects who were qualified with the above criteria. Further studies should also assess the effectiveness of anti-osteoporosis therapies for male subjects with high risks of fracture that are not identified to have low BMD or osteoporosis based on DXA criteria or prior fracture events.

## Conclusion

This systematic review confirms that bisphosphonates are connected with a decreased risk of vertebral fractures, nonvertebral fractures, and clinical fractures for male subjects with osteoporosis. Future studies will be required to further elucidate the role of nonbisphosphonates in treating fractures of osteoporosis subjects.

## Ethics Statement

This study is a systemic review with meta-analysis that did not involve experimental subjects or human tissues, and no sensitive data or private information were collected during the above study process.

## Author Contributions

JL and W-YY conceived and designed the study. The literature searches, study selection, critical appraisal, data extraction, and contacting authors of included studies for additional information were carried out by L-FZ, B-QP, M-HL, Z-TG, DZ, J-LZ, J-TL and J-KP. G-H L, YC, H-YC, H-TH, QL, Y-HH, J-HL, S-RH, MW and L-FZ conducted the interpretation and analysis of the relevant data. L-FZ, DG, W-XL, QW and JL drafted the paper. JL, A-HO and L-FZ further revised the text. The final version of this article was rechecked and approved by L-FZ, B-QP, G-HL, M-HL, YC, DG, H-YC, J-KP, H-TH, QL, Z-TG, Y-HH, DZ, J-LZ, S-RH, MW, J-TL, J-HL, W-XL, A-HO, QW, W-YY, and JL.

## Funding

This work was funded by the China Postdoctoral Science Foundation (No. 2018M633036), the Medical Science Research Foundation of Guangdong Province (No. B2019091), the National Natural Science Foundation of China (No. 81873314), the Project of Guangdong Provincial Department of Finance (No.[2014]157, No.[2018]8), Key Scientific Research Platforms and Research Projects of Universities in Guangdong Province (No. 2018KQNCX041), and the Science and Technology Research Project of Guangdong Provincial Hospital of Chinese Medicine (No. YK2013B2N19, YN2015MS15).

## Conflict of Interest Statement

The authors declare that the research was conducted in the absence of any commercial or financial relationships that could be construed as a potential conflict of interest.
