# Supplementary material for: Does Routine Anti-Osteoporosis Medication Lower the Risk of Fractures in Male Subjects? An Updated Systematic Review With Meta-Analysis of Clinical Trials
Source: Front Pharmacol. 2019 Aug 9;10:882. doi: 10.3389/fphar.2019.00882 (PMC6695469; doi:10.3389/fphar.2019.00882)
Supplement: Supplementary file 1 [file DataSheet_1.doc]

**Supplementary material (Figure S1-S13, and Table S1)**


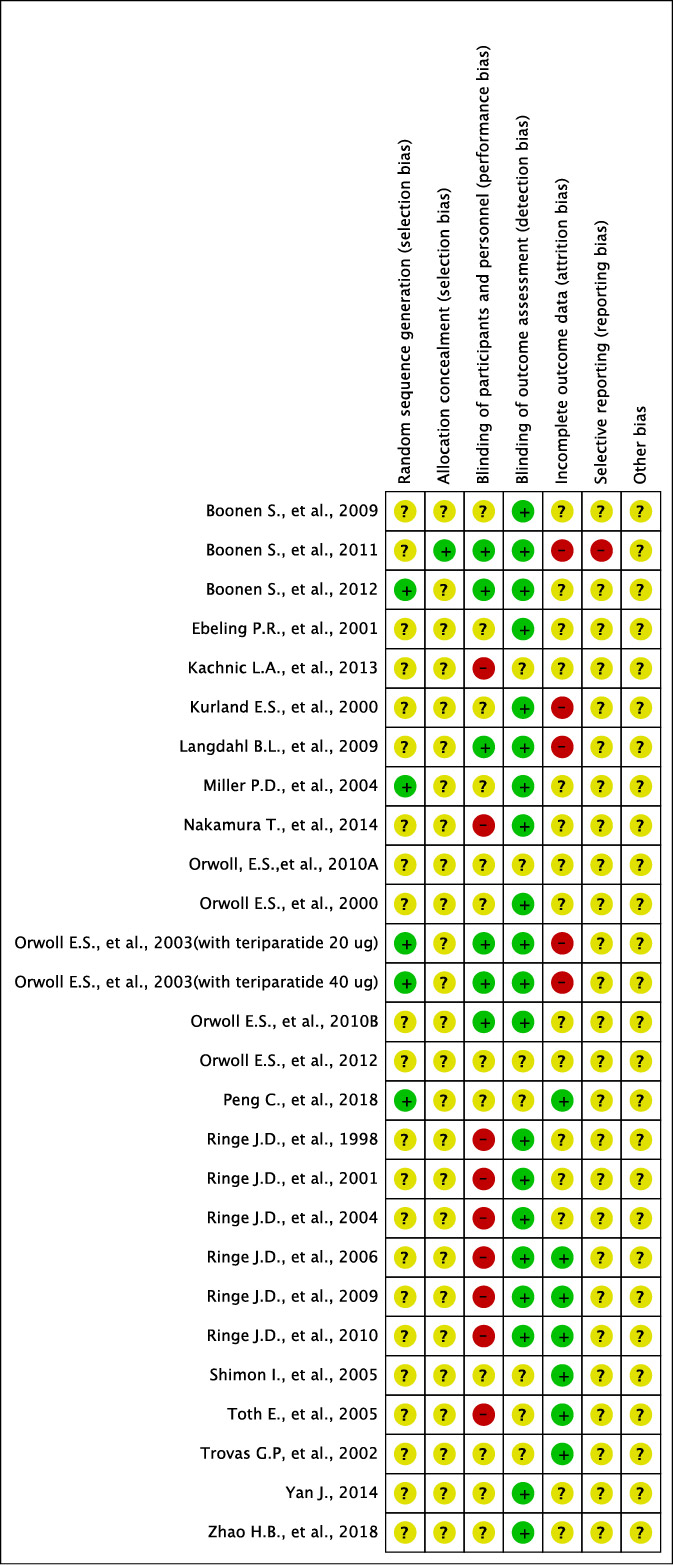


**Figure S1.** Assessment of risk of bias for the studies based on the Cochrane Handbook.


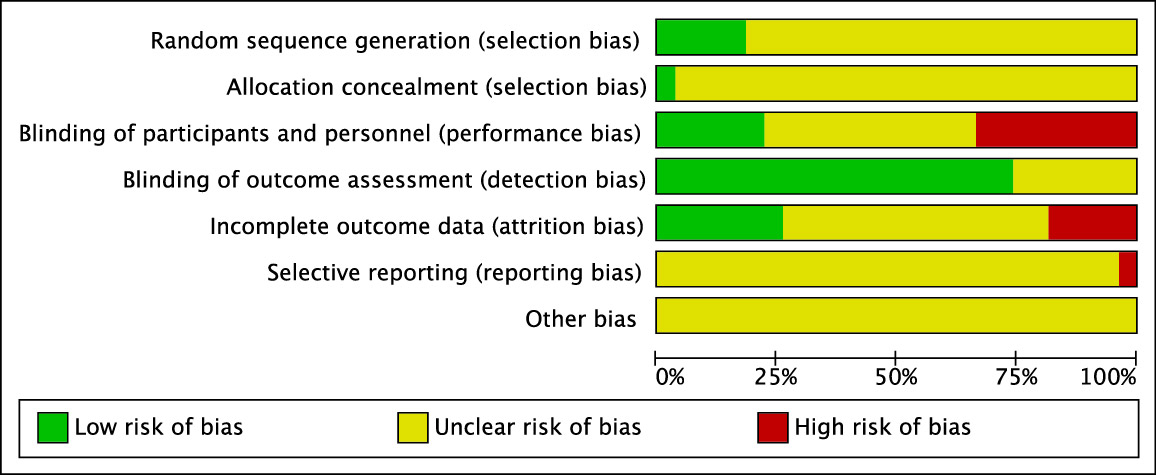


**Figure S2.** Risk of bias assessment for the studies included.


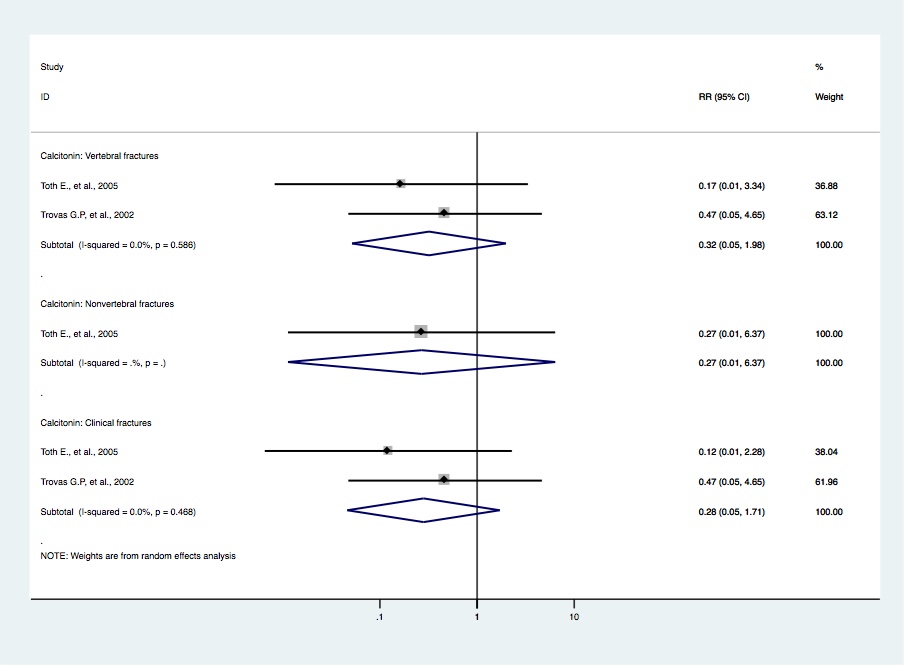


**Figure S3.** Forest plot of meta-analysis on calcitonin and osteoporotic fractures.

Notes: The size of diamond and box is positively proportional to the weight assigned to each study, and horizontal lines represent the 95%CI. RR = relative risk; CI = confidence interval.


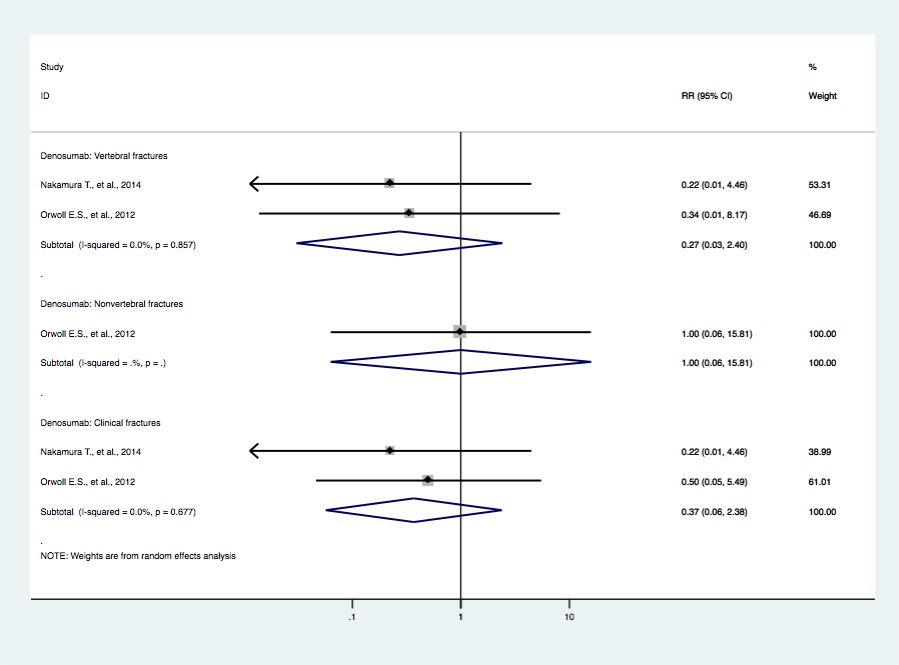


**Figure S4.** Forest plot of meta-analysis on denosumab and osteoporotic fractures.

Notes: The size of diamond and box is positively proportional to the weight assigned to each study, and horizontal lines represent the 95%CI. RR = relative risk; CI = confidence interval.


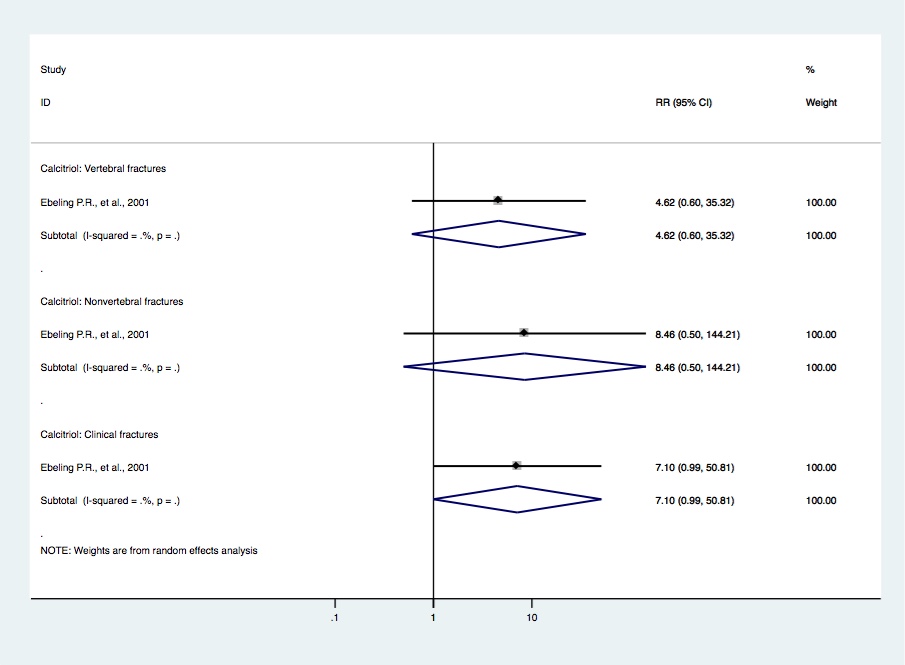


**Figure S5.** Forest plot of meta-analysis on calcitriol and osteoporotic fractures.

Notes: The size of diamond and box is positively proportional to the weight assigned to each study, and horizontal lines represent the 95%CI. RR = relative risk; CI = confidence interval.


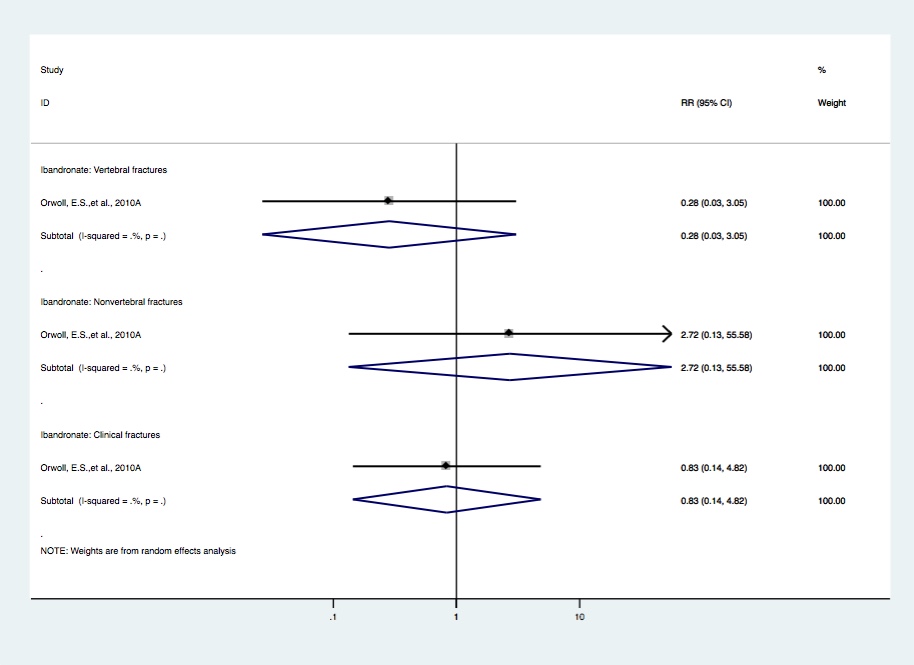


**Figure S6．** Forest plot of meta-analysis on cbandronate and osteoporotic fractures.

Notes: The size of diamond and box is positively proportional to the weight assigned to each study, and horizontal lines represent the 95%CI. RR = relative risk; CI = confidence interval.


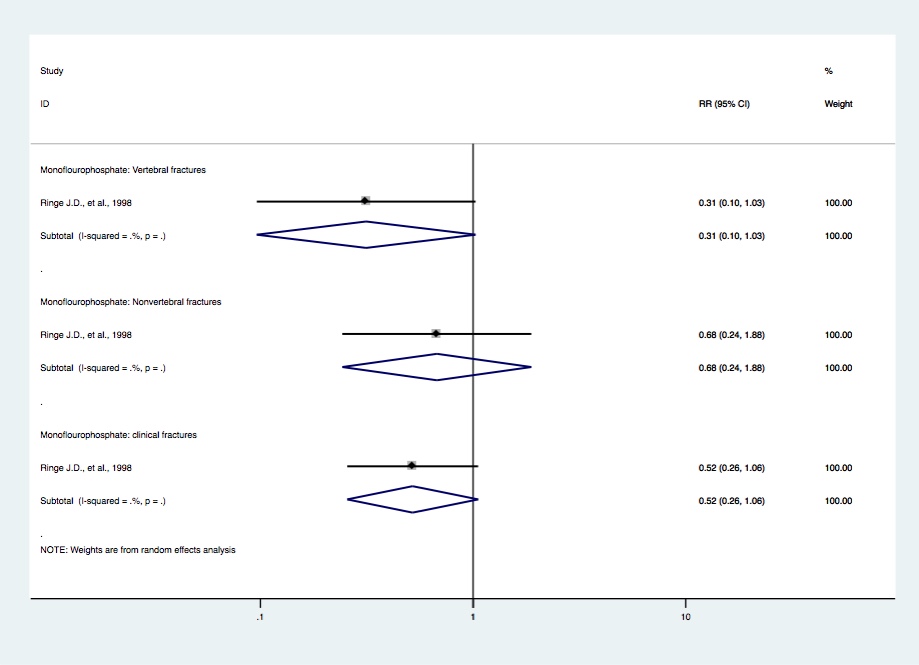


**Figure S7．** Forest plot of meta-analysis on monoflourophosphate and osteoporotic fractures.

Notes: The size of diamond and box is positively proportional to the weight assigned to each study, and horizontal lines represent the 95%CI. RR = relative risk; CI = confidence interval.


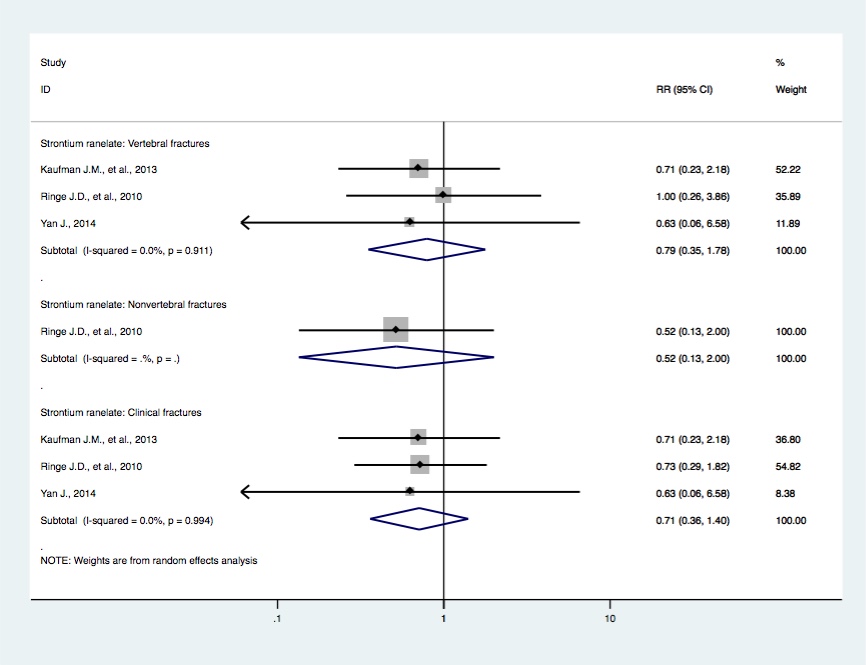


**Figure S8．** Forest plot of meta-analysis on strontium ranelate and osteoporotic fractures.

Notes: The size of diamond and box is positively proportional to the weight assigned to each study, and horizontal lines represent the 95%CI. RR = relative risk; CI = confidence interval.


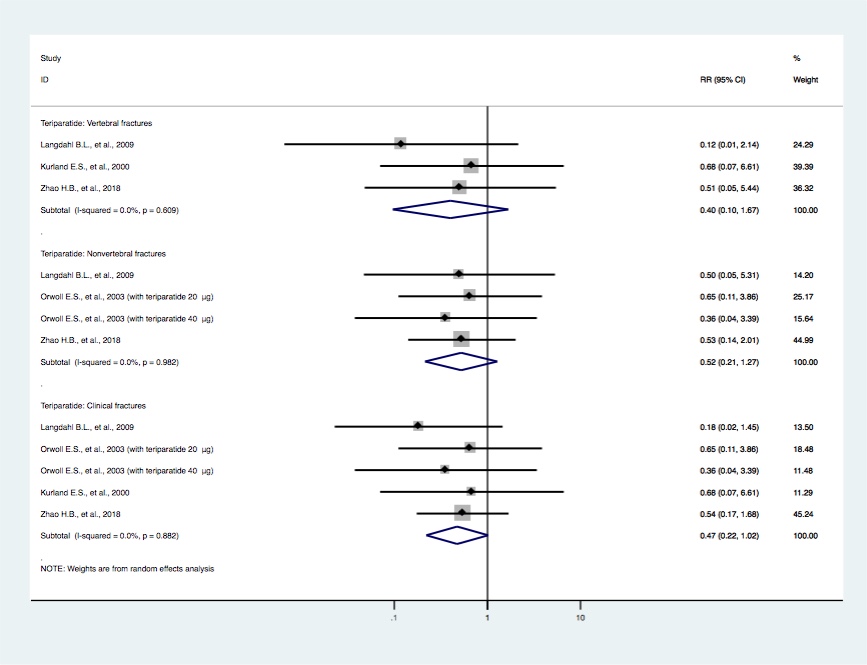


**Figure S9．** Forest plot of meta-analysis on teriparatide and osteoporotic fractures.

Notes: The size of diamond and box is positively proportional to the weight assigned to each study, and horizontal lines represent the 95%CI. RR = relative risk; CI = confidence interval.


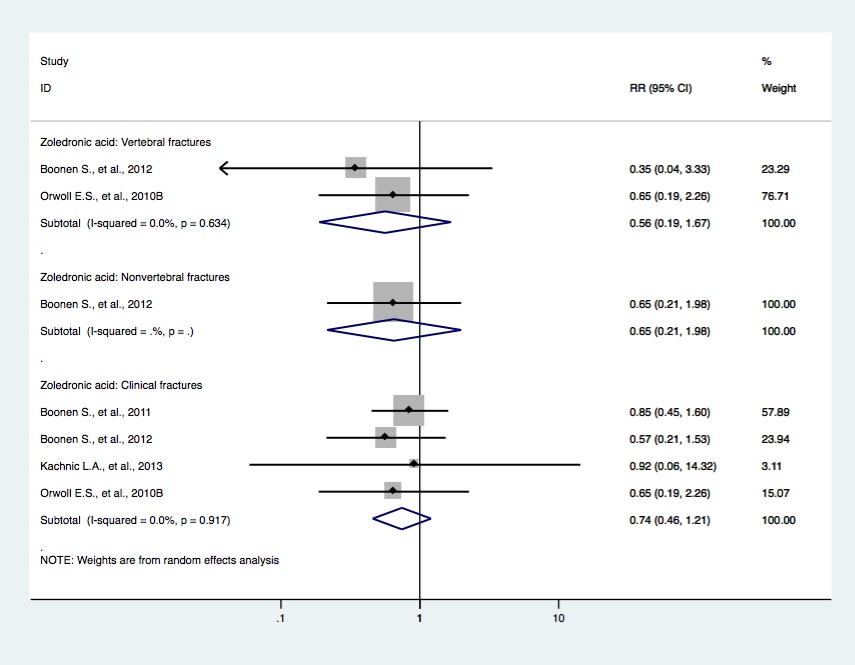


**Figure S10．** Forest plot of meta-analysis on zoledronic acid and osteoporotic fractures.

Notes: The size of diamond and box is positively proportional to the weight assigned to each study, and horizontal lines represent the 95%CI. RR = relative risk; CI = confidence interval.


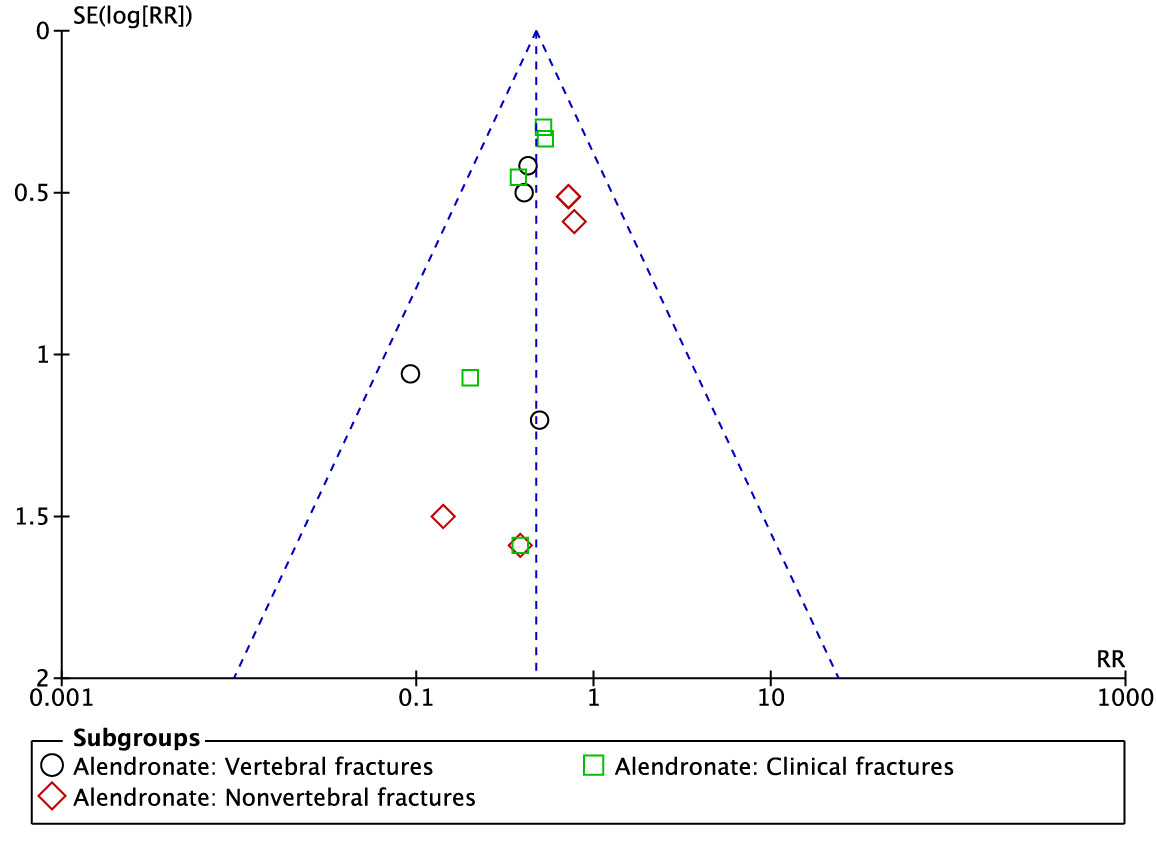


**Figure S11.** Funnel plots for the analysis of alendronate and osteoporotic fracture.

Notes: In the absence of publication bias, the points should be symmetrical about the vertical line at the pooled RRs. The reasonably symmetrical distribution suggests the absence of publication bias. RR = relative risk; SE = standard error.


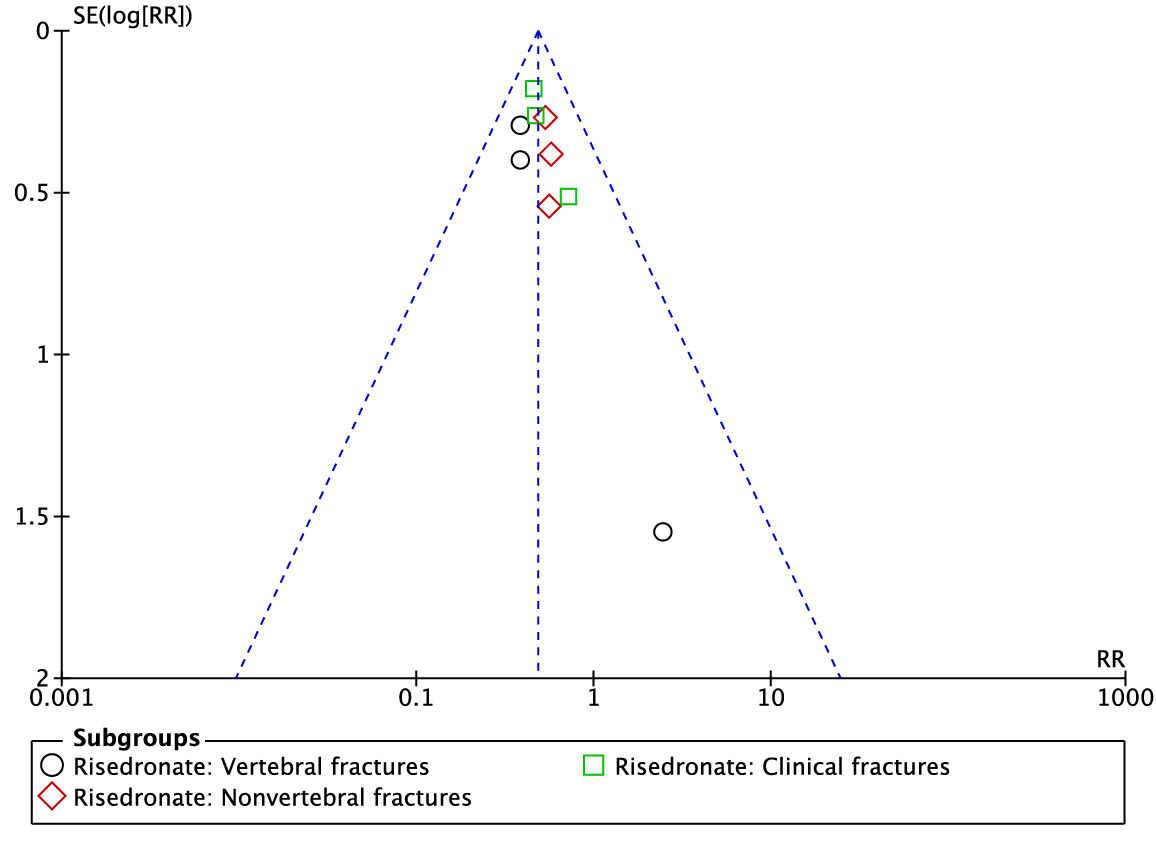


**Figure S12.** Funnel plots for the analysis of risedronate and osteoporotic fracture.

Notes: In the absence of publication bias, the points should be symmetrical about the vertical line at the pooled RRs. The reasonably symmetrical distribution suggests the absence of publication bias. RR = relative risk; SE = standard error.


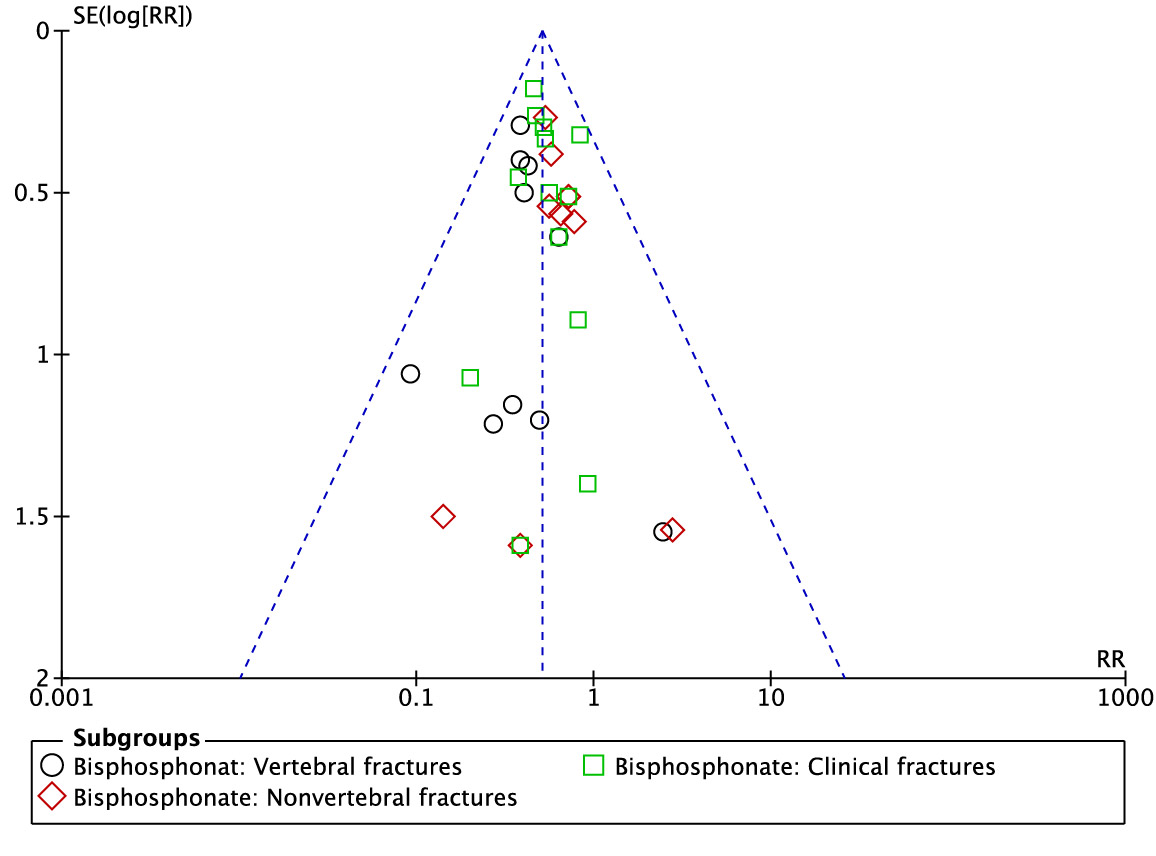


**Figure S13．**Funnel plots for the analysis of bisphosphonate and osteoporotic fracture.

Notes: In the absence of publication bias, the points should be symmetrical about the vertical line at the pooled RRs. The reasonably symmetrical distribution suggests the absence of publication bias. RR = relative risk; SE = standard error.

**Table S1.** A full electronic search strategy for the PubMed database.

| #1 osteoporosis  #2 osteopenia  #3 fracture  #4 bone density  5# bone mass  6# bone  7# bone disease  8# 1# OR 2# OR 3# OR 4# OR 5# OR 6# OR 7#  9# alendronate  10# risedronate  11# ibandronate  12# teriparatide  13# calcitriol  14# calcitonin  15# denosumab  16# monofluorophosphate  17# strontium ranelate  18# zoledronic acid  19# bisphosphonates  20# 10# OR 11# OR 12# OR 13# OR 14# OR 15# OR 16# OR 17# OR 18# OR 19#  21# 8# AND 20# |
| --- |
